# Avaliação de fadiga e fatores associados em pessoas submetidas à hemodiálise

**DOI:** 10.15649/cuidarte.2120

**Published:** 2021-10-06

**Authors:** Marinéia Albrecht Kickhöfel, Eda Schwartz, Lílian Moura de Lima Spagnolo, Aurélia Danda Sampaio, Tuany Nunes Cunha, Fernanda Lise

**Affiliations:** 1 Universidade Federal de Pelotas, Pelotas, RS, Brasil, E-mail: marineiakickhofel@gmail.com Autora para correspondência Universidade Federal de Pelotas Universidade Federal de Pelotas Pelotas RS Brazil marineiakickhofel@gmail.com; 2 Universidade Federal de Pelotas, Pelotas, RS, Brasil. E-mail: edaschwa@gmail.com Universidade Federal de Pelotas Universidade Federal de Pelotas Pelotas RS Brazil edaschwa@gmail.com; 3 Universidade Federal de Pelotas, Pelotas, RS, Brasil. E-mail: lima.lilian@gmail.com Universidade Federal de Pelotas Universidade Federal de Pelotas Pelotas RS Brazil lima.lilian@gmail.com; 4 Universidade Federal de Pelotas, Pelotas, RS, Brasil. E-mail: aurelia.sampaio@hotmail.com Universidade Federal de Pelotas Universidade Federal de Pelotas Pelotas RS Brazil aurelia.sampaio@hotmail.com; 5 Universidade Federal de Pelotas, Pelotas, RS, Brasil. E-mail: tuanynunes@hotmail.com Universidade Federal de Pelotas Universidade Federal de Pelotas Pelotas RS Brazil tuanynunes@hotmail.com; 6 Universidade Federal de Pelotas, Pelotas, RS, Brasil. E-mail: fernandalise@gmail.com Universidade Federal de Pelotas Universidade Federal de Pelotas Pelotas RS Brazil fernandalise@gmail.com

**Keywords:** Insuficiência Renal Crônica, Fadiga, Diálise Renal, Enfermagem., Renal Insufficiency, Chronic, Fatigue, Renal Dialysis, Nursing., Insuficiencia renal crónica, Fatiga, Diálisis renal, Enfermería.

## Abstract

**Introdução:**

o sintoma de fadiga é considerado incapacitante e afeta a qualidade de vida das pessoas com doença renal crônica, em tratamento de hemodiálise.

**Objetivo:**

avaliar a prevalência, intensidade e severidade do sintoma de fadiga em pessoas com doença renal crônica em tratamento hemodialítico.

**Materiais e Método:**

Estudo quantitativo descritivo, do qual participaram 335 pessoas em tratamento hemodialítico. Para a coleta dos dados foram utilizadas as escalas Visual Analog Scale” de 100mm, escala de severidade de fadiga e um questionário sociodemográfico. Foram analisadas as variáveis dependentes das escalas de fadiga, com as independentes de caracterização sociodemográfica e clínica.

**Resultados:**

dentificaram-se 58% do sexo masculino, idade entre 20-59 anos em 52,2%, predomínio de cor branca em 68,2%, 68,6% com baixa escolaridade, aposentadoria como principal renda em 55,7%, renda entre R$ 800,01 e R$1760,00 em 42,4%, a prevalência de fadiga foi de 63,0%, fadiga severa em viúvos (35,2%) e mulheres (27,6%), sendo que a principal comorbidade foi a Hipertensão Arterial Sistêmica (40,9%). A intensidade de fadiga teve o valor médio de 42,3(DP=32,2) e a severidade de fadiga teve valor médio de 35,8(DP=17,8).

**Conclusões:**

evidenciou-se que o fator socioeconômico e as comorbidades influenciam no sintoma de fadiga em mulheres, viúvos e idosos. O uso de instrumentos de avaliação pode contribuir para melhorar a abordagem da Enfermagem, e em consequência, o bem-estar e a qualidade de vida destas pessoas.

## Abstract (en)

**Introduction:**

The symptom of fatigue is considered incapacitating and affects the quality of life of people with chronic kidney disease, undergoing hemodialysis treatment. Objective: to assess the prevalence, intensity, and severity of the symptoms of fatigue in people with chronic kidney disease in hemodialysis treatment.

**Materials and Methods:**

Descriptive quantitative study, in which 335 in hemodialysis treatment participated. For the data collection, were used the 100mm Visual Analog Scale”, the scale of severity of the fatigue, and a sociodemographic questionnaire. Were analyzed variable dependent on the fatigue scale, with the independent variables of sociodemographic and clinical characterization.

**Results:**

58% were identified as male, aged between 20-59 years old in 52.2%, predominance of White color in 68.2%, 68.6% with low schooling, retirement as main income in 55.7%, yields between R$ 800.01 and R$ 1760.00 in 42.4%, with a prevalence of fatigue were 63.0%, severe fatigue in viúvos (35.2%) and women (27.6%), being that the main comorbidities of Systemic Arterial Hypertension (40.9%). At the intensity of fatigue, it has a mean value of 42.3 (DP = 32.2) and at the severity of fatigue, it has a mean value of 35.8 (DP = 17.8).

**Conclusions:**

We evidenced that the socioeconomic factor in comorbidities influences the symptoms of fatigue in women, widowers, and the elderly. The use of assessment instruments can contribute to help us address the sickness, and consequent- ly, or improve the quality of life of these people.

## Resumen

**Introducción:**

: el síntoma de la fatiga se considera incapacitante y afecta a la calidad de vida de las personas con enfermedad renal crónica en tratamiento de hemodiálisis.

**Objetivo:**

: evaluar la prevalencia, intensidad y gravedad del síntoma fatiga en personas con enfermedad renal cróni- ca en tratamiento de hemodiálisis.

**Materiales y métodos:**

: Estudio descriptivo cuantitativo, en el que participaron 335 personas en tratamiento de hemodiálisis. Para la recogida de los datos se utilizó la Visual Analog Scale” de 100mm, escala de gravedad de la fatiga y un cuestionario sociodemográfico. Se analizaron las variables dependientes de las escalas de fatiga, con las in- dependientes de caracterización sociodemográfica y clínica.

**Resultados:**

: 58% eran hombres, 52,2% tenían entre 20-59 años, 68,2% eran predominantemente blancos, 68,6% tenían bajo ni- vel de estudios, 55,7% tenían la jubilación como ingreso principal, 42,4% tenían ingresos entre R$ 800,01 y R$ 1760,00, la prevalencia de la fatiga fue de 63,0%, fatiga severa en viudos (35,2%) y mujeres (27,6%), y la principal comorbilidad fue la hipertensión arterial sistémica (40,9%). La intensidad de la fatiga tuvo un valor medio de 42,3 (DP=32,2) y la gravedad de la fatiga tuvo un valor medio de 35,8 (DP=17,8).

**Conclusiones:**

: se evidenció que el factor socioeconómico y las comorbilidades influyen en el síntoma de fatiga en mujeres, viudos y ancianos. El uso de instrumentos de evaluación puede contribuir a mejorar el abordaje de la enfermedad y, en con- secuencia, el bienestar y la calidad de vida de las personas.

## Introdução

A Doença Renal Crônica (DRC) é caracterizada por alterações heterogêneas ou anormalidades que afetam a estrutura e a função renal, com redução progressiva da filtração glomerular por, pelo menos, três meses(1). Sua ocorrência é considerada um problema de saúde pública em todo o mundo. De acordo com o Censo Brasileiro de Diálise de 2018 a prevalência estimada é de 640 pacientes por milhão da população (pmp) em diálise, sendo a Hemodiálise (HD) a Terapia Renal Substitutiva (TRS) mais empregada em 92% dos usuários, predomínio do sexo masculino 58%, faixa etária entre 45-64 anos (41,5%), e com mais de 65 anos (35%)(2).

Durante o percurso da DRC, a fadiga é considerada um sintoma incapacitante, com diversos distúrbios clínicos e neurológicos, difícil de definir e estudar, pois trata de uma entidade distinta e altamente subjetiva(3), tornando-se o principal sintoma de impacto negativo na qualidade de vida (QV). Além disso, por estar associada a comorbidades, tais como depressão, anemia, alterações na qualidade do sono, além de fatores demográficos, tornando alta sua prevalência, na maioria dos casos permanecendo subdiagnosticada e subtratada, aumentando os eventos cardiovasculares e mortalidade em pacientes com DRC(4).

As pessoas com DRC que desenvolvem fadiga apresentam maior prevalência de xerodermia (pele seca), prurido (coceira), mialgia (dor muscular), artralgia (dor nas articulações), osteodinia (dor nos ossos), síndrome das pernas inquietas, dispneia (falta de ar), sensação de tristeza e ansiedade, dificuldade de concentração e para se excitar, em relação aos não fadigados, pode gerar limitações de ordem física, social e emocional, com sérias repercussões na vida do paciente e de sua família(5).

Além disso, a fadiga pode ser mais acentuada em pacientes com DRC em TRS, devido às longas distâncias e vias de difícil acesso percorridas para acessar os STRS e, ainda, por vezes, pela indisponibilidade de transporte, gerando cansaço físico e psicológico, devido ao fato de os STRS estarem centralizados nos grandes centros econômicos dos estados(6). Portanto, faz-se necessário olhar para esta temática, tendo em vista que a fadiga gera o impacto negativo na QV de pessoas com DRC em tratamento hemodialítico. Sendo assim, esse estudo objetivou avaliar a prevalência, intensidade e severidade do sintoma de fadiga em pessoas com Doença Renal Crônica em tratamento hemodialítico.

## Materiais e Método

Estudo quantitativo descritivo, seguiu o checklist STROBE para estudos observacionais. Recorte da macropesquisa “Atenção à saúde nos serviços de terapia renal substitutiva da Metade Sul do Rio Grande do Sul” e “Empoderamento e autonomia para pessoas dos serviços de terapia renal da metade sul do Rio Grande do Sul”. Realizado nos municípios de Pelotas, Rio Grande, São Lourenço do Sul, Alegrete e Uruguaiana, caracterizando a metade Sul do Rio Grande do Sul e Fronteira Oeste do Rio Grande do Sul e, também, por serem os municípios desta região os que disponibilizam o Serviço de Terapia Renal Substitutiva (STRS).

A amostra foi composta de 335 pacientes em tratamento hemolítico, dos 364 cadastrados nos STRS, 29 foram excluídos por ausência no serviço no momento da coleta ou por não terem condições de responder, no período de 2016 e 2017 e àqueles que participaram da macropesquisa. Foram critérios de seleção da amostra da macropesquisa, ser usuário cadastrado no serviço e estar presente no momento da coleta de dados; ter mais de 18 anos; capacidade de comunicar-se verbalmente; capacidade cognitiva preservada. Para a construção do banco de dados foi realizada dupla digitação no *software* EpiDATA 3.1, e, posteriormente, os dois bancos foram pareados e ajustadas as inconsistências, em seguida as variáveis foram analisadas com o *software Stata* 13.0.

Para mensurar a ocorrência de fadiga, utilizaram-se às variáveis da escala analógica visual Visual Analog Scale”- VAS de 100mm(7) e a escala de severidade de fadiga(3) por meio de análise estatística descritiva, com distribuição de frequências absolutas e relativas e medidas de tendência central (médias e medianas) e de dispersão (desvio-padrão) das variáveis. Para identificar a intensidade e a severidade da fadiga, avaliou-se a intensidade de fadiga com a média e o desvio-padrão obtidos na variável escala analógica de fadiga. Já, para a severidade da fadiga, obteve-se um escore composto das respostas das nove questões que compõem a escala, a qual tem opções de resposta com notas que variam de 1 a 7. O escore final obtido na escala variou de 9 a 63, sendo considerado um indicativo de escores de fadiga a partir de 28. Resultados entre 28 e 39 caracterizam fadiga leve, de 40 a 51 moderada e de 52 a 63 grave(8).

Para cada variável analisada foi expresso o valor observado na amostra (n) e o percentual (%). Adotou-se o *p-*valor<0,05 para assumir a hipótese de associação entre as variáveis sociodemográficas e clínicas com o sintoma de fadiga. Para tanto, foram comparadas as variáveis dependentes, escala analógica de fadiga e escala de severidade de fadiga e independentes, sexo, estado civil, idade, cor da pele, fonte de renda, renda, comorbidades, mora sozinho, anos de estudo e município pelo teste não paramétrico de *Kruskal-Wallis* e *Mann-Whitney*(9).

Foram seguidos os princípios éticos da Resolução 466/2012(10) e recebeu aprovação do Comitê de Ética em Pesquisa da Faculdade de Enfermagem com o parecer número 1.386385 e CAAE 51678615300005316.

## Resultados

Identificou-se a prevalência de 63,0% de fadiga entre os entrevistados com base nos resultados obtidos com os 335 participantes. A intensidade de fadiga verificada teve o valor médio de 42,3(DP=32,2) variando de 0 a 99, com mediana de 45. Quanto à severidade de fadiga, responderam a essa variável 303 dos 335 entrevistados, obtendo-se o valor médio de 35,8(DP=17,8) e variando de 9 a 63 com mediana de 37.

As características sociodemográficas das pessoas em hemodiálise na metade sul do Rio Grande Sul e os escores de severidade do sintoma de fadiga são apresentados na Tabela 1.

**Tabela 1:** Distribuição das características sociodemográficas e clínicas dos pacientes em hemodiálise na metade sul do Rio Grande do Sul pela severidade de fadiga, Pelotas, RS, 2017, N=303.

Características dos entrevistados (n)	Ausência		Leve		Severidade da fadiga				Total N=303			
					Moderada		Severa					**Valor de *p***
	n	%	n	%	n	%	N	%	N	%	
Sexo (302)				
Feminino	40	31,5	26	20,5	26	20,5	35	27,6	127	42,0	0,09*
Masculino	71	40,6	32	18,3	36	20,6	36	20,6	175	58,0	
Estado civil (303)											
Casado	68	39,8	32	18,6	36	21,1	35	20,5	171	56,4	0,19**
Solteiro	28	40,0	13	18,6	12	17,1	17	24,3	70	23,1	
Divorciado/ Separado	6	24,0	8	32,0	5	20,0	6	24,0	25	8,3	
Viúvo	10	27,0	5	13,5	9	24,3	13	35,2	37	12,2	
Idade (299)											
18-19	1	33,3	0	0	2	66,7	0	0	3	1,0	0,27**
20-59	66	42,3	26	16,7	32	20,5	32	20,5	156	52,2	
60 a mais	45	32,1	30	21,4	28	20,0	37	53,6	140	46,8	
Cor da pele (302)											
Branco	68	33,0	42	20,4	46	22,3	50	24,3	206	68,2	0,08*
Não Branco	44	45,8	16	16,7	15	15,6	21	21,9	96	31,8	
Fonte de renda (300)											
Emprego	2	22,2	2	22,2	2	22,2	3	33,4	9	3,0	0,77**
Renda familiar	11	32,4	7	20,6	8	23,5	8	23,5	34	11,3	
Benefício	35	38,9	16	17,8	17	18,9	22	24,4	90	30,0	
Aposentadoria	63	37,7	33	19,8	33	19,8	38	22,7	167	55,7	
Renda (302)											
Menor 880,00	17	37,8	3	6,6	8	17,8	17	37,8	45	14,9	0,16**
880,01 a 1760,00	49	38,3	20	15,6	30	23,4	29	22,7	128	42,4	
1760,01 a 2640,00	27	32,5	19	22,9	17	20,5	20	24,1	83	27,5	
Maior que 2,640,01	19	41,3	15	32,6	7	15,2	5	10,9	46	15,2	
Comorbidades (291)											
Nenhuma	36	38,7	26	28,0	17	18,3	14	15,0	93	32,0	0,16**
HAS&	44	37,0	17	14,3	26	21,8	32	26,9	119	40,9	
DM#	14	50,0	3	10,7	5	17,9	6	21,4	28	10,0	
HAS e DM	15	29,4	9	17,6	13	25,5	14	27,5	51	17,5	
Mora sozinho (303)											
Não	101	38,1	52	19,6	51	19,3	61	23,0	265	87,5	0,22*
Sim	11	28,9	6	15,8	11	28,9	10	26,4	38	12,5	
Anos de estudo (303)											
Nãoestudou	5	29,4		7	41,2		2	11,8		3	17,6	17	5,6	0,95**
Até 9	77	37,0		37	17,8		46	22,1		48	23,1	208	68,6	
10-16	30	38,5		14	18,0		14	18,0		20	25,6	78	25,7	
Município (302)											
Pelotas	40	30,1		26	19,5		33	24,8		34	25,6	133	44,0	0,10**
Rio Grande	23	43,4		7	13,2		8	15,1		15	28,3	53	17,5	
São Lourenço do Sul	7	26,9		7	26,9		4	15,4		8	30,8	26	8,6	
Alegrete	27	51,9		8	15,4		7	13,5		10	19,2	52	17,2	
Uruguaiana	14	36,9		10	26,3		10	26,3		4	10,5	38	12,6	

&HAS (Hipertensão Arterial Sistêmica) # DM (Diabetes Melitus) *Teste de *Mann-Whitney;* **Teste de Kurskalwallis *** salário mínimo brasileiro em 2016

Fonte: Elaboração própria a partir do banco de dados dos projetos “Atenção à saúde nos serviços de terapia renal substitutiva da Metade Sul do Rio Grande do Sul” e “Empoderamento e autonomia para pessoas dos serviços de terapia renal da metade sul do Rio Grande do Sul”.

Dentre os participantes, 58,0% (n=175) eram do sexo masculino, porém, a maior concentração de fadiga ocorreu entre as mulheres 68,6% (n=87), sendo que 27,6% (n=35) apresentaram fadiga severa. Já, em relação ao estado civil, com 303 entrevistados o predomínio foi de pessoas casadas com 56,4%(n=171), destas 39,8% (n=68) não apresentavam fadiga e 41,6%(n=71) apresentavam fadiga entre moderada 21,1%(n=36) e severa 20,5%(n=35), 40,0%(n=70) eram solteiros e sem fadiga, entre divorciados ou separados 32,0%(n=25) houve fadiga leve e 35,2%(n=37) dos viúvos apresentaram fadiga severa. Em relação à idade (n=299), 52,2%(n=156) tinham entre 20- 59 anos, sendo que 42,3%(n=66) sem fadiga e 41,0%(n=64) apresentaram fadiga, distribuindo- se igualmente entre moderada e severa. Já, entre os participantes com mais de 60 anos, 53,6%(n=37) apresentaram fadiga severa.

A cor da pele (n=302) prevalente foi a branca com 68,2%(n=206) e 67%(n=138) destes apresentaram fadiga entre leve, moderada e severa. Dos 31,8%(n=96) não brancos, 45,8%(n=44) não apresentaram fadiga. A fonte de renda destas pessoas (300) concentra-se principalmente na aposentadoria por idade 55,7%(167) e destes, 62,3 %(104) apresentam fadiga entre leve, moderada e severa. A renda (302) é para 42,4%(128) de 880,01 a 1760,00 e destes, 61,7%(79) apresentam fadiga, sendo 46,1%(59) entre fadiga moderada 23,4%(30) e severa 22,7%(29).

Quanto às comorbidades (n=291), 40,9%(n=119) dos participantes apresentaram Hipertensão Arterial Sistêmica, dos quais 63%(n=75) apresentavam fadiga, sendo 26,9 %(n=32) severa.

Possuíam companhia em sua moradia 87,5%(n=265) dos participantes, dos quais 61,9 %(n=164) apresentaram fadiga, sendo severa em 23%(n=61) dos casos. Já dos 12,5%(n=38) que moravam sozinhos, 71,1%(n=27) apresentaram fadiga e destes, 28,9%(n=11) com fadiga moderada. Os participantes apresentavam escolaridade baixa, até nove anos 68,6%(n=208) e destes, 63%(n=131) apresentaram fadiga, sendo a severa a mais prevalente com 23,1%(n=48). Dentre os que tiveram escolaridade acima de dez anos, 61,6%(n=44) apresentaram fadiga, sendo 25,6%(n=20) com fadiga severa, e este grupo também foi o que apresentou a maior porcentagem de pessoas com ausência de fadiga 38,5%(n=30).

Em relação ao município, os participantes foram oriundos de Pelotas em 44%(n=133) e destes, 69,9%(n=93) apresentaram fadiga, concentrando-se entre moderada 24,8%(n=33) e severa 25,6%(n=34). Outros municípios que demonstraram igualdade quase completa em quantidade de participantes foram Rio Grande 17,5%(n=53) e Alegrete com 17,2%(n=52), no entanto, entre eles existe uma diferença, pois, enquanto em Rio Grande, 56,6%(n=30) apresentaram fadiga, sendo severa em 28,3%(n=15), em Alegrete, 51,9%(n=27) não demonstraram fadiga. Já, em São Lourenço e Uruguaiana que são serviços com menor número de participantes, ambos apresentaram altos índices de fadiga, em São Lourenço, 8,6%(n=26) participantes apresentaram 73,1% de fadiga, sendo severa em 30,8%(n=8) e Uruguaiana com 12,6%(n=38) participantes, 63,1%(n=24) apresentaram fadiga, entre leve e moderada, distribuindo-se igualmente com 26,3%(n=10) cada um deles.

Ao analisar todas as variáveis, nenhuma teve significância estatística na distribuição pela severidade de fadiga.

## Discussão

Diante da subjetividade do sintoma de fadiga e do impacto que este causa sobre a qualidade de vida e saúde da pessoa em tratamento hemodialítico, nota-se a importância de identificar tal sintoma entre pessoas com DRC, posto que a existência do sintoma de fadiga foi encontrada em alguns estudos(5),(11) nas diferentes modalidades de terapia renal de substituição. Neste estudo, identificou-se existência de fadiga nos participantes, assim como em estudos internacionais que também encontraram alta existência deste sintoma, variando de 44,7%(12) a 100%(13) dos participantes.

A principal comorbidade encontrada neste estudo associadaà DRCequeinfluencianosintomadefadiga em pessoas fazendo tratamento hemodialítico foi a Hipertensão Arterial Sistêmica (HAS), sendo dentre os fadigados a maior prevalência com fadiga severa, o que corrobora os achados do estudo que elucidou a questão de que a HAS e Diabetes Mellitus (DM) são as principais doenças de base da DRC no Brasil(2), sendo responsáveis por quase metade dos indivíduos que estão em tratamento dialítico, sendo o DM a principal etiologia de base para a DRC(13); já as comorbidades que influenciam diretamente na fadiga(14)são os diagnósticos de anemia(15), ansiedade e depressão(5).

O gênero é outro fator que exerce influência no sintoma de fadiga, para o qual as mulheres são mais acometidas(16), o que corrobora este estudo, o qual identificou um maior número de homens, embora a concentração maior fadiga apresentou-se nas mulheres. Considera-se que essa porcentagem de mulheres esteja associada à função hormonal e, muitas vezes, pela dupla jornada de trabalho, com emprego formal, cuidado doméstico, cuidado com filhos, além do tratamento são alguns fatores que possivelmente justifique este achado. Da mesma forma, pessoas de etnia branca são mais propensas a desenvolver o sintoma de fadiga, o que pode estar relacionado com a maior quantidade de pessoas brancas em tratamento hemodialítico, uma vez que no estado do Rio Grande do Sul a predominância da população é de cor branca(17). Embora esperava-se encontrar uma maior quantidade de negros, pois possuem maior probabilidade de desenvolver hipertensão e diabetes(18), que são preditivos de DRC.

Evidenciou-se neste estudo que a principal fonte de renda foi a aposentadoria por idade em que os participantes ganhavam entre R$ 880,01 a R$ 1760,00 equivalentes a aproximadamente $220,00 a $440,00 na época da coleta do estudo, a renda concentrava-se entre um e dois salários mínimos, o que também pode ser relacionado com o comprometimento das atividades do trabalho, devido às limitações e ao tempo gasto para a realização das modalidades terapêuticas (hemodiálise), propiciandoodesempregoe, assim, ummaiorimpactosocioeconômico. Osfatores socioeconômicos foram referidos no estudo que apontou o baixo nível de escolaridade como o principal fator que influencia na renda e adesão ao tratamento(16), o que está em conformidade com este estudo, em que os participantes possuíam baixo índice de escolaridade, com até 9 anos de estudo. O grau de escolaridade, também, se destaca na promoção de saúde, pois a partir dele pode-se avaliar a qualidade das informações prestadas e o nível de compreensão demonstrado pelo paciente em relação às recomendações feitas pelo profissional da saúde, sobretudo no que se refere aos cuidados em saúde, hábitos de vida e o tratamento(19). Quanto maior a escolaridade, maior é o acesso às informações e melhoria das condições econômicas, desse modo, pessoas com maior grau de escolaridade exercem atividades que exigem menor esforço físico, e por isso o impacto da doença em suas atividades é menor, além de assimilar mais adequadamente as informações e colaborar com a adesão ao tratamento(19).

Identificou-se que pessoas que não moram sozinhas estão em maior número dos participantes, porém as que moram sozinhas foram as mais fadigadas, prevalecendo entre moderada e severa, indo ao encontro do estudo que revelou que baixo apoio familiar piorou o sintoma de fadiga, enquanto um alto nível de apoio social diminuiu o nível de fadiga(14). Assim como o número de pessoas na residência demonstrou ser fator auxiliar, pois a existência de um companheiro e/ou residir com a família pode contribuir para a manutenção dos cuidados no domicílio, bem como nas atividades diárias. O que foi evidenciado neste estudo em que os casados representaram menor índice de fadiga, portanto, o estado civil tem influência no sintoma de fadiga, pois ter um companheiro melhora o enfrentamento das adversidades, tal e qual o apoio familiar e de amigos age como alavanca para manter o equilíbrio, estimula as mudanças dos hábitos e promove determinados comportamentos que melhoram, por sua vez, a saúde geral e a qualidade de vida(20).

Evidenciou-se também por meio deste estudo, que os viúvos e as pessoas com mais de 60 anos apresentaram fadiga severa, fator possivelmente relacionado com a falta do companheiro/a e a necessidade do apoio da família no caso dos idosos, pois eles tendem a ser mais dependentes para a realização das atividades diárias, portanto, o apoio familiar impacta em melhor qualidade de vida dessas pessoas(4),(14), (18),(21).

Na variável relacionada com o município, identificou-se que praticamente metade dos participantes do estudo realizavam TRS em Pelotas, e com maior índice de fadiga, possivelmente pelo fato de Pelotas ser uma cidade de grande porte na metade sul do Rio Grande do Sul, posto que em municípios de médio e grande porte, a quantidade e intensidade dos laços afetivos são menores (ou muitas vezes inexistentes), diferentemente do que acontece em cidades pequenas, onde a intensidade dos relacionamentos interpessoais são bem maiores(22). Além de fatores como violência, falta de segurança, trânsito e outros estressores que propiciam o isolamento social e o desencadeamento de transtornos de humor nas grandes cidades e nos ambientes agitados das grandes metrópoles. Evidenciamos isto em relação ao município de Alegrete em que a maioria dos participantes não apresentaram o sintoma de fadiga, o que, também, pode estar relacionado com a cultura local, o tamanho da cidade e o Índice de Desenvolvimento Humano (IDHM) da cidade, pois, em 2010, o IDHM de Alegrete era de 0,740, o que situa o referido município na faixa de Desenvolvimento Humano Alto (IDHM entre 0,700 e 0,799). A dimensão que mais contribui para o IDHM do município é Longevidade, com índice de 0,849, seguida de Renda, com índice de 0,720, e de Educação, com índice de 0,664(23).

Os serviços de hemodiálise de Pelotas ainda possuem outro fator que influencia o alto índice de fadiga, uma vez que recebe pacientes advindos de outros municípios da região. O município mais distante de Pelotas fica localizado a 250 km, com duração em média de viagem as cidades de três horas, afetando a qualidade de vida das pessoas que necessitam percorrer longas distâncias, com transporte precário, gerando desgaste físico e emocional para receber o tratamento hemodialítico(6). Tratamento que possibilita a melhoria dos sintomas, porém esse recurso é ininterrupto, tornando-se cansativo e repetitivo, uma vez que a rotina contínua e duradoura das sessões impõe ao paciente a interrupção das suas atividades diárias, a dedicação de quatro horas, três vezes por semana, diante disto, as intervenções lúdicas, se bem conduzidas, podem ser satisfatoriamente aceitas e aproveitadas em casos de tratamento duradouro, tornam-se aliadas para suavizar o peso do processo crônico, pois é durante as sessões de hemodiálise que as pessoas com DRC compartilham um período de convivência e de diálogo, uma oportunidade para expressar emoções e compartilhar medos e temores com outros pacientes e profissionais da saúde, favorecida pelas ações de promoção da saúde, melhorando a auto-estima, depressão e necessidade de convívio social, o que pode impactar positivamente na qualidade de vida destes(24).

Além das atividades lúdicas favorecerem as ações de promoção da saúde, podemos também mencionar o uso da tecnologia como um potencial de integração inteligente entre os serviços de saúde e a continuidade dos cuidados, aumentando o espectro de possibilidades de atuação e alcançando, simultaneamente, um número incontável de pessoas(25) permitido um autocuidado mais eficaz, assim proporcionando melhor qualidade de vida as pessoas em tratamento hemodialítico.

## Conclusão

Baseando-se na análise dos dados concluiu-se que 63% dos participantes apresentavam o sintoma de fadiga, com intensidade variando de 0 a 99 e mediana de 45, já a severidade variando de 9 a 63, com mediana de 37. Ao correlacionar com aspectos sociodemográficos e clínicos, evidenciou-se que as mulheres, viúvos, idosos e hipertensos apresentaram maior predomínio de fadiga severa e que o fator psicossocial e socioeconômico influencia no sintoma de fadiga, assim como o apoio da família e o fato de não morar sozinho pode aumentar a qualidade de vida de pessoas em tratamento hemodialítico.

As ações de promoção de saúde favorecem o entendimento da doença, proporcionam momentos de troca e permitem aos profissionais da saúde conhecer melhor o perfil da clientela atendida para o planejamento de cuidados visando uma assistência de qualidade. Assim enfermeiros, como a equipe multiprofissional que atuam na atenção a pessoa com DRC, devem procurar identificar e minimizar os sintomas de fadiga, avaliando seus exames laboratoriais, fazendo o diagnóstico precoce da fadiga, por meio da aplicação das escalas disponíveis para tal, com efeitos simples e de baixo custo. Da mesma forma podem fazer uso do genograma e ecomapa, para conhecer a sua rede de apoio social e de apoio, promovendo maior qualidade de vida, melhorando seu autocuidado e tarefas do dia a dia e, consequentemente, aumentando sua autoestima e o bem-estar.

O estudo tem limitações de regionalidade, por ter sido aplicado em uma área especifica do Rio Grande do Sul, sendo assim, os resultados podem ser diferentes em demais regiões do Brasil.
